# Study on the Effect of Sorghum Flour Particle Size on the Storage Quality of Leavened Pancakes

**DOI:** 10.3390/foods13121934

**Published:** 2024-06-19

**Authors:** Xueqin Li, Jingru Tian, Fei Xu, Yingguo Lv

**Affiliations:** 1 College of Food Science and Engineering, Henan University of Technology, Zhengzhou 450001, China; xueqinl216@sina.cn (X.L.); tianjingr@163.com (J.T.); yingguo_lv@126.com (Y.L.); 2 Henan Province Wheat-Flour Staple Food Engineering Technology Research Centre, Zhengzhou 450001, China; 3 Food Laboratory of Zhongyuan, Luohe 462001, China

**Keywords:** sorghum flour, particle size, leavened pancake, storage quality

## Abstract

Pancakes prepared with sorghum flour possess a high nutritional value, yet their quality is unstable and prone to degradation during storage. This instability can be attributed to the particle size of coarse cereal powder, which significantly influences the quality of flour products during storage. In this study, changes in the quality of these pancakes, prepared with varying particle sizes of sorghum flour, were meticulously analyzed during cold storage using advanced instruments such as a texture analyzer, nuclear magnetic resonance spectrometer, differential scanning calorimeter, X-ray diffractometer, and Fourier transform infrared spectrometer. Findings revealed that the hardness of leavened pancakes significantly increased over time. After a refrigeration period of 7 days, the hardness of wheat flour leavened pancakes increased by 56.60%. However, with a decrease in the particle size of sorghum flour, the increase in hardness diminished, thereby delaying the aging process of the pancakes. As the storage duration was extended, moisture migration within the pancakes occurred, and the sorghum flour pancakes with a smaller particle size exhibited a reduced moisture change rate, indicating an enhanced water holding capacity. In comparison to their wheat flour counterparts, sorghum flour leavened pancakes exhibited a substantial reduction in retrogradation enthalpy and crystallinity. The inclusion of sorghum flour effectively inhibited amylopectin recrystallization, thus slowing down the aging process of the pancakes. This inhibitory effect was more evident with decreasing sorghum flour particle sizes. Fourier transform infrared data indicated no significant alterations in absorption peaks across various wavelengths during cold storage. Although starch short-range orderliness increased with storage time, the use of sorghum flour with smaller particles reduced the degree of short–range orderliness in starch molecules throughout the cold storage period. Sorghum flour with a smaller particle size can inhibit water migration and amylopectin recrystallization, which subsequently delays pancake aging and enhances its quality stability during storage.

## 1. Introduction

As a traditional characteristic Chinese flour product, the leavened pancake boasts a long history and unique flavor, endearing it to consumers and securing its significant place in Chinese food culture. Comprised of wheat flour and yeast which have undergone mixed fermentation, it offers a soft texture, ease of consumption, and a rich wheat aroma, complemented by the distinct scent of fermentation. Additionally, the baked leavened pancake possesses a distinctive baked flavor. Nevertheless, similar to all starchy foods, the quality alterations that occur during storage impact the edible quality of the leavened pancake and curtail its shelf life. Primarily, these alterations are associated with phenomena such as starch retrogradation, moisture migration, and the interaction between starch and gluten proteins [1,2]. With the extension of storage time, amylopectin regenerates and forms a recrystallized structure, which leads to the hardening of texture, the weakening of flavor, and a decrease in the nutritional value and digestibility of flour products [3]. During the storage process of fermented flour products, due to the existence of the water gradient, the internal moisture migrates to the outside and eventually volatilizes, resulting in a drying and hardening of flour products [4]. At the same time, the interaction between gluten proteins and starch molecules influences the aging of leavened pancakes. A robust gluten network decelerates the aging of starch, whereas weak gluten may hasten starch recrystallization, thereby intensifying the aging of flour products. This occurs because the hydrophilicity of the protein is augmented, leading to a more pronounced interaction between the protein and the starch granules, which facilitates the formation of starch recrystallization [5]. Xiang et al. [6] found that during the storage process, the retrogradation enthalpy (heat absorption required for starch to return from a gelatinized state to its original ecology, ΔH) of steamed bread showed a trend of increasing first fast and then slow, but with an increase in a wheat bran addition, ΔH gradually decreased, indicating that wheat bran could inhibit the recrystallization of amylopectin in steamed bread, thus delaying the aging of steamed bread. Qiao et al. [7] showed that during the storage of fresh brown rice noodles at 37 °C and 25 °C, the deep bound water content decreased, the weakly bound water content increased first and then decreased, and the free water content decreased first and then increased. The relative proportion of various forms of water in fresh brown rice noodles changes due to moisture migration, resulting in changes in its quality.

The concept of healthy eating has taken deep root in the collective consciousness of people, leading to an increased popularity among consumers of flour products that incorporate whole grain flour into wheat flour. Owing to its rich nutritional content, distinctive functional properties, high yield, and consistent product quality, sorghum flour is extensively utilized in staple flour–based foods. Yousif et al. [8] showed that the addition of sorghum flour to wheat flour reduced the rapidly digestible starch content of sorghum bread, and increased the polyphenol content and antioxidant capacity, indicating that sorghum flour may reduce the impact on human postprandial blood glucose levels and the risk of chronic diseases. However, the high mechanical strength and water absorption capacity of the crude fiber in the sorghum seed coat make it challenging for traditional crushing technologies to refine the crude fiber in sorghum flour effectively. This results in a rough taste, uneven surface, and poor quality of sorghum products [9]. Studies have shown that the morphological structure and size difference of grain particles are related to the characteristics of dough and the quality of flour products [10]. Qin et al. [11] studied the effect of crushing particle size on the quality of gluten–free rice bread. The findings indicated that the specific volume of gluten–free rice bread, produced from rice flour with a larger particle size, was notably reduced compared to that made from rice flour with a smaller particle size. Consequently, the crumb structure of the bread was coarser, leading to an unsatisfactory quality of the gluten–free rice bread. Hence, it is crucial to investigate the impact of varying grain flour particle sizes on the quality of flour–based products by crushing the grain particles to different extents.

In this study, alterations in hardness, moisture content, moisture migration, starch thermal properties, starch crystallinity, and short–range molecular order within starch were meticulously examined during the refrigerated storage of leavened pancakes with sorghum flour of varying particle sizes. The extent of pancake aging and the impact of sorghum flour granularity on the aging process were also elucidated, thereby furnishing a theoretical framework for extending the shelf life and enhancing the storage quality of leavened pancakes.

## 2. Materials and Methods

### 2.1. Materials

Special flour (14.10% moisture, 0.48% ash, 9.68% protein, 0.65% fat, 16.10% amylose, 56.10% amylopectin, 2.89% fiber) was supplied by Jinyuan Flour Co., Ltd. (Zhengzhou, China). Sorghum flour (9.98% moisture, 0.40% ash, 8.47% protein, 0.55% fat, 25.20% amylose, 46.43% amylopectin, 8.91% fiber) was supplied by Beichun Agricultural Products Development Co., Ltd. (Mudanjiang, China). High activity dry yeast was obtained from Angel Yeast Co., Ltd. (Yichang, China)

### 2.2. Preparation of Sorghum Flour with Different Particle Sizes

Commercially available sorghum flour was put into an ultrafine grinder (WF–18, Wenzhou Dingli Medical Device Co., Ltd., Wenzhou, China) for crushing. The commercially available sorghum powder was ground for 10 s and 30 s respectively to obtain sorghum powder samples with median particle sizes of about 30 μm and 20 μm.

### 2.3. Particle Size Determination

The particle size distributions of three kinds of sorghum flour were determined by a laser particle size analyzer (BT–9300S, Dandong Baite Instrument Co., Ltd., Dandong, China) under the wet method.

### 2.4. Preparation and Storage Technology of Sorghum Flour Leavened Pancake

Wheat flour was replaced by sorghum flour with different particle sizes at a ratio of 15%, and mixed and set aside. A total of 200 g mixed flour and a certain amount of water were weighed, and 2.0 g yeast was dissolved in the water to make it melt. The dissolved yeast water and flour were poured into a needle–type dough machine (JHMZ–200, Beijing Dongfu Jiuheng Instrument Technology Co., Ltd., Beijing, China), and the dough was mixed for 2 min to form a smooth dough. Then the dough was placed in a constant temperature and humidity oven (LHS–50CL, Shanghai Yiheng Technology Co., Ltd., Shanghai, China), and the fermentation humidity was 80%, the fermentation temperature was 35 °C, and the fermentation time was 60 min. The dough was over–pressed 20 times (JMTD–168/140 test noodles machine, Beijing Dongfu Jiuheng Instrument Technology Co., Ltd., Beijing, China), the bubbles in the dough were discharged, and the dough was rolled into a long strip, which was then divided into dough pieces with weights of 90 g. Then, a circular biscuit with a diameter of 10 cm and a thickness of 1 cm was made by a circular biscuit press. The biscuit was placed in the constant temperature and humidity oven for 30 min, and the biscuit was baked in the oven (T3–L326B, Guangdong Midea Kitchen Appliance Manufacturing Co., Ltd., Foshan, China). The baking temperature was set at 190 °C and the baking time was 11 min. After cooling for 60 min, the quality of the leavened pancake was determined. The prepared leavened pancake samples were placed in a sealed bag and stored in a refrigerator (BCD–225AG, Beijing Hisense Electric Co., Ltd., Beijing, China) at 4 °C. The relevant indicators were measured after 1, 3, 5, and 7 days.

### 2.5. Hardness

The hardness was determined by reference to Sheikholeslami et al. [12], with slight modifications. The hardness of the sorghum leavened pancakes were determined by a TA–XT Plus texture analyzer (TA–XT Plus, Stable Micro System, London, UK). The sample was put on the stage. The TPA (texture profile analysis) mode was selected for testing, and the probe of the p/35 model was selected. The measurement parameters were set as follows: pre–test speed 3 mm/s, test speed 1 mm/s, post–test speed 1 mm/s, and compression ratio 50%.

### 2.6. Moisture Content

According to the direct drying method in the GB/T 5009.3-2016 National Food Safety Standard Determination of Moisture in Food, the moisture content in the leavened pancakes was determined [13].

### 2.7. Moisture Distribution

The method of He et al. [14] was referred to, with minor modifications. A low–field nuclear magnetic resonance analyzer (Micro MR–CL–I, Suzhou Neumay Electronic Technology Co., Ltd., Suzhou, China) was used to determine the moisture distribution of the leavened pancakes. A certain quantity of leavened pancake was weighed and put into a 25 mm diameter nuclear magnetic tube. The spin relaxation time test was performed using the Carr–Purcell–Meiboom–Gill pulse sequence. The test parameters were set as follows: sampling interval time TW = 2000 ms, echo time TE = 0.1 ms, echo number NECH = 3000, and cumulative number NS = 16.

### 2.8. Thermal Properties of Starch

The method of Wang et al. [15] was referred to, with minor modifications. The thermodynamic properties of the leavened pancakes were determined by differential scanning calorimetry analyzer (DSC) (DSC250, TA Instruments, New Castle, DE, USA). The powder of the freeze–dried leavened pancake sample was weighed at 2–3 mg, and deionized water was added according to the ratio of 1: 3 sample to water. After reaching equilibrium at room temperature overnight, the blank crucible was used as a control for thermodynamic determination. The samples were heated at 5 °C/min from 20 °C to 120 °C.

### 2.9. Starch Crystallinity

The hardness was determined by reference to Ahmed et al. [16], with slight modifications. The leavened pancake samples, refrigerated for 1, 3, 5, and 7 days, were freeze–dried and ground through 100 sieves. The crystallinity of the samples was determined by X-ray diffraction analyzer (XRD) (Rigaku Mini Flex 600, Rigaku Corporation, Akishima, Japan). The scanning area was set to 4–45 °, and the scanning speed was 4 °/min. The relative crystallinity of the samples was calculated by MDI Jade 6 treatment.

### 2.10. Short–Range Ordering of Starch Molecules

The method of Jiang et al. [17] was referred to, with minor modifications. The freeze–dried leavened pancake sample powder was placed in a Fourier infrared spectrometer (PerkinElmer Spectrum TWO, Perkin Elmer, Waltham, MA, USA) and scanned in absorbance mode. The scanning range was 400–4000 cm^−1^, the number of scans was 32 times, and the resolution was 4 cm^−1^. The baseline correction of the spectral data was performed using OMNIC 9.2 software. The data in the range of 800–1200 cm^−1^ were selected for Fourier self–deconvolution processing, and the half–peak width and enhancement factor were set to 19 cm^−1^ and 1.9 cm^−1^, respectively.

### 2.11. Statistical Analysis

All experiments were repeated 3 times, and the results were expressed as mean ± standard deviation. Excel 2010 software was used for data processing, SPSS 20.0 was used for one–way analysis of variance, and Duncan’s test was used for significance analysis at the level of *p* < 0.05. Origin 2021 software was used for plotting.

## 3. Results and Discussion

### 3.1. Particle Size Distribution of Sorghum Flour

The particle size distribution of flour is a key factor affecting the quality of flour and its products [18]. The commercially available sorghum flour was named SF1, and the sorghum flours after different times of ultrafine grinding were named SF2 and SF3. The particle size distribution of the three kinds of sorghum flour are shown in Table 1. D_50_ and D_(4, 3)_ are important parameters that reflect the average particle size of the samples. It can be seen from Table 1 that D_50_ and D_(4, 3)_ of the sorghum flour decreased significantly after ultrafine grinding. The median particle size of SF1 was 53.37 μm, and D_50_ was reduced to 31.93 μm and 20.55 μm after ultrafine grinding, indicating that ultrafine grinding can effectively reduce the particle size of sorghum flour.

### 3.2. Changes in Hardness of Sorghum Flour Leavened Pancakes during Storage

**Figure 1:**
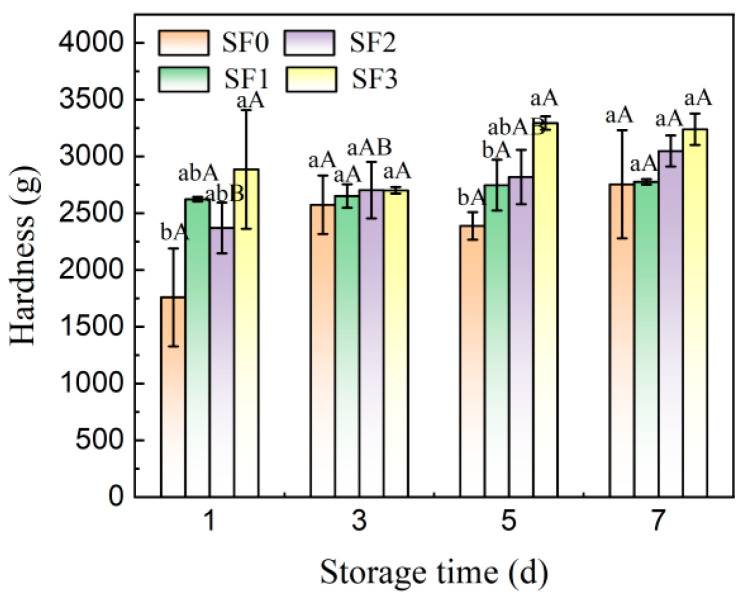
Changes in hardness of sorghum flour leavened pancakes with different particle sizes during storage. SF0: wheat flour leavened pancake; SF1: sorghum flour leavened pancake with SF1 added; SF2: sorghum flour leavened pancake with SF2 added; SF3: sorghum flour leavened pancake with SF3 added. Different lowercase letters indicate significant differences between different treatments at the same storage time (*p* < 0.05). Different capital letters indicate significant differences in the same treatment at different storage times (*p* < 0.05).

**Figure 2:**
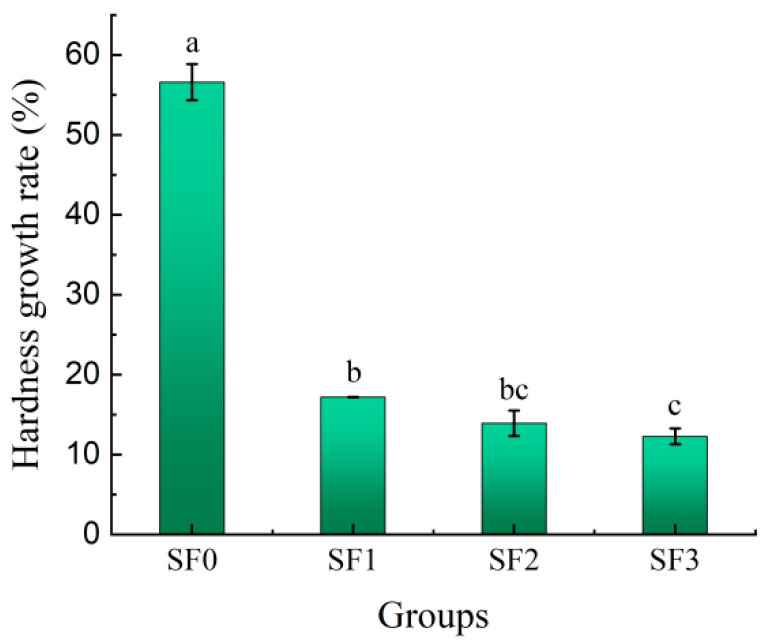
Hardness growth rates of sorghum flour leavened pancakes with different particle sizes stored for 7 days. Different lowercase letters indicate significant differences between different treatments at the same storage time (*p* < 0.05).

The leavening of pancakes is accompanied by water loss and starch retrogradation during storage, making change in hardness the most direct indicator for assessing the extent of pancake aging. The evolution of hardness in sorghum flour leavened pancakes over storage duration is depicted in Figure 1 and Figure 2. It can be seen from the figures that the hardness of the leavened pancakes increased significantly with the increase in storage time. After 7 days of cold storage, the hardness value of the wheat flour leavened pancake reached 2753.99 g, and the growth rate was 56.60%, indicating that the quality of the wheat flour leavened pancake deteriorated rapidly at 4 °C and the storage resistance was poor. The hardness growth rates of sorghum flour leavened pancakes with different particle sizes were 17.19%, 13.92%, and 12.27%, respectively, indicating that sorghum flour with smaller particle sizes delayed the aging rate of leavened pancakes and was beneficial for maintaining the quality of leavened pancakes during storage. This is because as the particle size of sorghum flour decreases, the water holding capacity of leavened pancakes increases, reducing the migration of moisture, thereby reducing the aging rate of leavened pancakes. In addition, the dietary fiber in sorghum flour does not gelatinize during the baking process, and fills in gaps between the starch networks to prevent the association between starch chains, which can delay the aging of the leavened pancake to a certain extent [19].

### 3.3. Changes in Moisture Content of Sorghum Flour Leavened Pancakes during Storage

**Figure 3:**
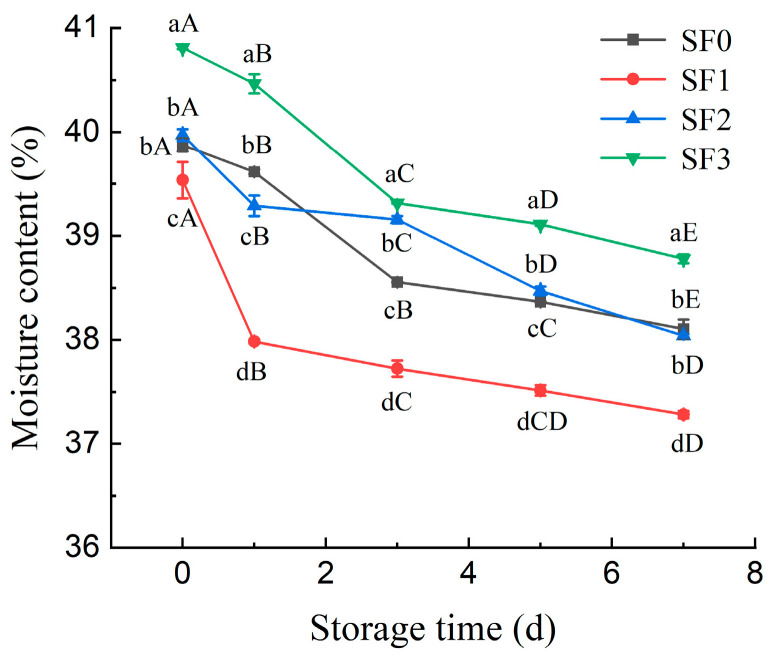
Changes in moisture content of sorghum flour leavened pancakes with different particle sizes during storage. SF0: wheat flour leavened pancake; SF1: sorghum flour leavened pancake with SF1 added; SF2: sorghum flour leavened pancake with SF2 added; SF3: sorghum flour leavened pancake with SF3 added. Different lowercase letters indicate significant differences between different treatments at the same storage time (*p* < 0.05). Different capital letters indicate significant differences in the same treatment at different storage times (*p* < 0.05).

During storage, moisture content is the primary factor influencing the quality of leavened pancakes. The aging rate of these pancakes is directly affected by their moisture content, with higher moisture levels resulting in a slower aging process. Figure 3 shows the change in moisture content of sorghum flour leavened pancakes during storage.

It can be seen from Figure 3 that the moisture content of the SF1 leavened pancake was lower than that of the SF2 leavened pancake and the SF3 leavened pancake at day 0 of storage. This is because the water absorption of the mixed flour after adding sorghum flour is different, and the amount of water added when making leavened pancakes is different, which makes the moisture content of the leavened pancakes different. With the extension of storage time, the moisture content of the leavened pancakes gradually decreased. At 0–3 days, the rate of decline was faster, and the rate of decline slowed down at 3–7 days. On the 7th day, the moisture content of the four groups of leavened pancakes decreased by 4.44%, 5.69%, 4.97%, and 4.83%, respectively, which indicated that the sorghum flour with smaller particle sizes showed better water holding capacity and delayed the loss of water during the storage of leavened pancakes.

### 3.4. Changes in Moisture Distribution of Sorghum Flour Leavened Pancakes during Storage

In the process of storage, the internal moisture of leavened pancakes migrates to the surface and evaporates by diffusion, resulting in the hard and poor quality of leavened pancakes [20]. Moisture migration has an important influence on the quality of leavened pancakes.

**Figure 4:**
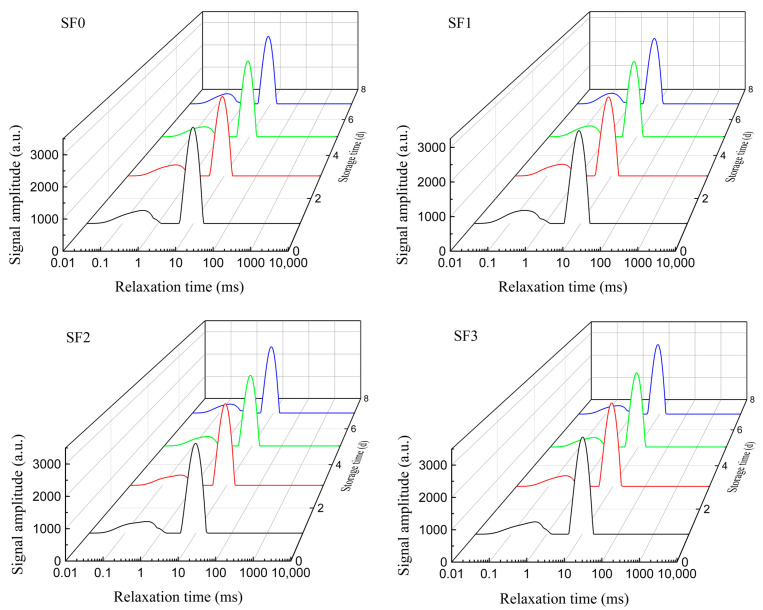
_1_HT^2^ relaxation curves of sorghum flour leavened pancakes with different particle sizes during storage.

The moisture distribution and migration of leavened pancakes during storage can be detected by low–field nuclear magnetic technology, so as to provide a reference for improving the water retention capacity and aging resistance of leavened pancakes [21]. Figure 4 shows the _1_HT^2^ relaxation curves of sorghum flour leavened pancakes with different particle sizes during cold storage. As shown in the figure, the leavened pancakes mainly contain two states of water during storage, which are T_21_ and T_22_. T_21_ with a shorter transverse relaxation time represents deep bound water, and T_22_ with a longer transverse relaxation time represents weakly bound water. The smaller the T_2_ value, the tighter the water binding and the better the water holding capacity.

The changes in the water relaxation time and water binding state of sorghum flour leavened pancakes with different particle sizes during cold storage are shown in Table 2. It can be seen from the table that with the extension of storage time, the T_21_ and T_22_ of the leavened pancakes decreased significantly and shifted to a shorter relaxation time. This is because the gelatinized starch formed an orderly crystalline structure during storage, and the water molecules combined with amorphous starch moved to the crystalline region, which reduced the fluidity of water molecules [22,23]. The percentages of T_21_ and T_22_ changes in the four groups of leavened pancakes were 36.84%, 42.42%, 41.46%, 36.96% and 18.84%, 18.76%, 18.74%, 12.94%, respectively. This shows that with the decrease in sorghum flour particle size, the ability to control the outflow of weakly bound water from the leavened pancake system is stronger, which can slow down the water loss.

A_21_ and A_22_ represent the percentage of deep bound water and weakly bound water in the total water content, respectively. It can be seen from the table that, with the extension of storage time, the A_21_ of the leavened pancakes showed a decreasing trend as a whole, and the A_22_ showed an increasing trend as a whole, indicating that during the cold storage process, the tightly bound deep bound water in the leavened pancakes gradually changed to weakly bound water that could flow more easily. This is due to the extension of the cold storage time, during which the growth in ice crystals in the leavened pancakes leads to the weakening of the gluten network structure, which reduces the water holding capacity of the leavened pancakes [24]. This observation aligns with the alteration in moisture content of the leavened pancakes during storage. The percentage of A_21_ and A_22_ changes in the four groups of leavened pancakes were 4.05%, 6.86%, 3.58%, 3.29% and 1.26%, 1.13%, 1.07%, 0.94%, respectively, indicating that the smaller particle size of sorghum flour can inhibit moisture migration, thereby delaying the aging of sorghum flour leavened pancakes.

### 3.5. Changes in Thermal Characteristics of Starch of Sorghum Flour Leavened Pancakes during Storage

During the storage of leavened pancakes, starch molecules re–form a double helix structure through hydrogen bonding. This phenomenon is called starch retrogradation, and starch retrogradation is an important reason for the aging of leavened pancakes [25]. Using DSC to determine the retrogradation enthalpy of the leavened pancake samples during storage can effectively reveal the degree of amylopectin recrystallization and clearly evaluate the aging degree of the leavened pancakes [26].

**Figure 5:**
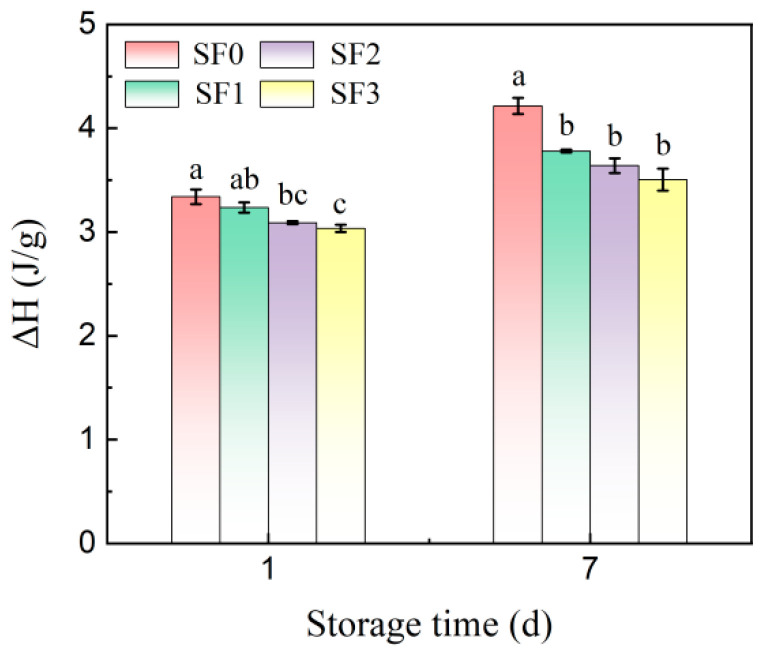
Changes in retrogradation enthalpy of sorghum flour leavened pancakes with different particle sizes during storage. Different lowercase letters indicate significant differences between different treatments at the same storage time (*p* < 0.05).

The retrogradation enthalpy values of sorghum flour leavened pancakes with different particle sizes stored at 4 °C for 1 day and 7 days are shown in Figure 5. From Figure 5, it can be seen that during storage, the retrogradation enthalpy of the wheat flour leavened pancake in the control group increased from 3.33 J/g to 4.21 J/g, while the retrogradation enthalpy of sorghum flour leavened pancakes was significantly lower than that of the wheat flour leavened pancake, indicating that the addition of sorghum flour can significantly inhibit the recrystallization of amylopectin and effectively delay the aging of leavened pancakes, and with the decrease in sorghum flour particle size, this inhibition is more obvious. Studies have shown that starch retrogradation and moisture migration are the main reasons for the aging of flour products [27], indicating that sorghum flour with smaller particle sizes can bind to water molecules, inhibit the loss of water during leavened pancake storage, hinder the formation of double helix structures, and further inhibit the recrystallization of starch, thus delaying the aging of leavened pancakes. The study of Wang et al. [25] showed that buckwheat shells with smaller particle sizes could delay the aging of amylopectin during bread storage.

In order to better reflect the change in retrogradation enthalpy of leavened pancakes during storage, the Avrami equation was introduced to fit the starch aging kinetics, and the parameters are shown in Table 3. The Avrami exponent n represents different nucleation modes. The larger the *n* value, the slower the nucleation rate.

It can be seen from the table that the *n* value of the leavened pancake system is greater than 1, indicating that recrystallization in the system is continuously occurring during the later storage period [28]. Compared with the wheat flour leavened pancake, sorghum flour leavened pancakes have a smaller *n* value, but with the decrease in sorghum flour particle sizes, the *n* value gradually increases from 1.026 to 2.380, indicating that the smaller particle sizes of sorghum flour reduce the nucleation rate of starch recrystallization during storage of leavened pancakes, delaying the aging process of starch [29]. The k value is the starch retrogradation rate constant. It can be seen from the table that as the particle size of sorghum flour decreases, the k value of the leavened pancakes gradually decreases to 0.043 and 0.032, indicating that the smaller particle sizes of sorghum flour leavened pancakes have a slower starch retrogradation rate and higher quality stability. This is consistent with the change in moisture content and hardness.

### 3.6. Changes in Starch Crystallinity of Sorghum Flour Leavened Pancakes during Storage

**Figure 6:**
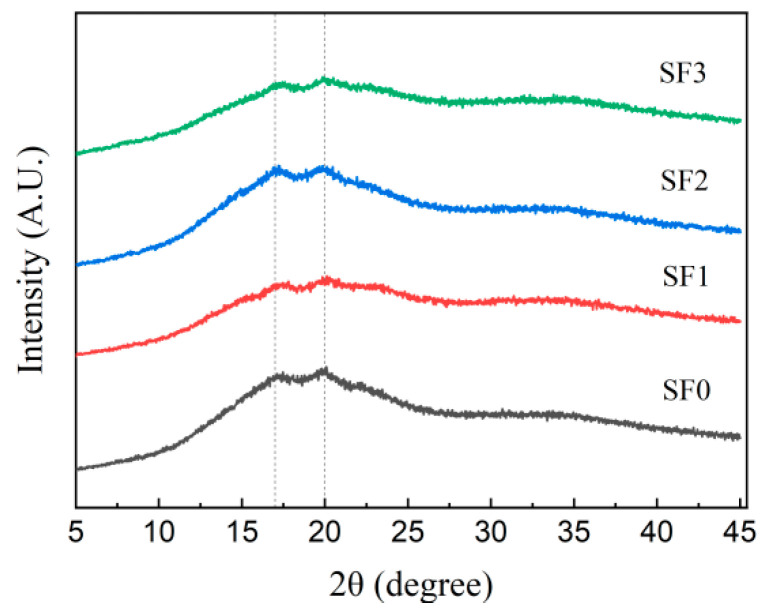
X-ray diffraction patterns of sorghum flour leavened pancakes with different particle sizes stored at 4 °C for 7 days.

XRD is the most widely used analysis method for evaluating the crystal structure of starch, which can be used to analyze the aging degree of starch [30]. Figure 6 shows the XRD diffraction pattern of different leavened pancakes stored at 4 °C for 7 days. It can be found that all samples have obvious diffraction peaks at the diffraction angles of 17° and 20°, which represent the stable β–type crystal structure formed during starch retrogradation and the V–type crystal structure formed by the interaction of fatty acids, phospholipids, and amylose, respectively [31,32]. It shows that the retrogradation of amylopectin in the leavened pancakes during storage causes the aging of the leavened pancakes. The relative crystallinity is the percentage of the above two peak areas to the total area, which can represent the different degrees of recrystallization of amylopectin in the stored leavened pancakes [33].

**Figure 7:**
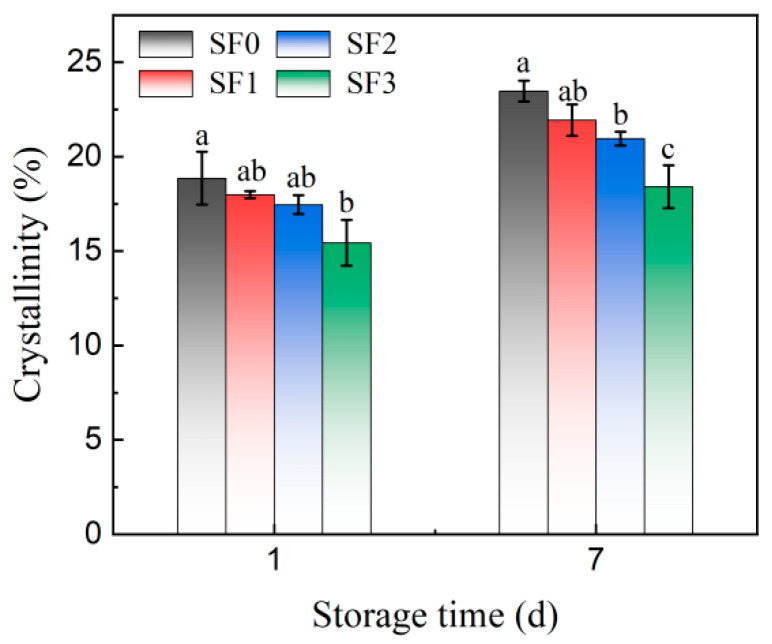
Changes in starch crystallinity of sorghum flour leavened pancakes with different particle sizes during storage. Different lowercase letters indicate significant differences between different treatments at the same storage time (*p* < 0.05).

The effect of sorghum flour on the crystallinity of leavened pancakes during storage is shown in Figure 7. It can be seen from Figure 7 that, after 7 days of storage, the crystallinity of all samples increased significantly, and the crystallinity of the four groups of leavened pancakes increased by 26.56%, 21.96%, 20.35%, and 19.24%, respectively. The addition of sorghum flour reduced the growth rate of the crystallinity of the leavened pancakes, and with the decrease in the particle sizes, this reduction effect was more obvious, indicating that sorghum flour could delay the aging of leavened pancakes by inhibiting the retrogradation of amylopectin. This may be due to the fact that sorghum flour with smaller particle sizes inhibits the diffusion and migration of moisture and inhibits starch recrystallization by interacting with starch and protein. This is consistent with the change in moisture content and the results of DSC analysis. The study of Gary et al. [34] also showed that the softness of bread was higher when fewer water molecules were involved in the recrystallization process of amylopectin.

### 3.7. Changes in Short–Range Order of Starch Molecules during Storage of Sorghum Flour Leavened Pancakes

**Figure 8:**
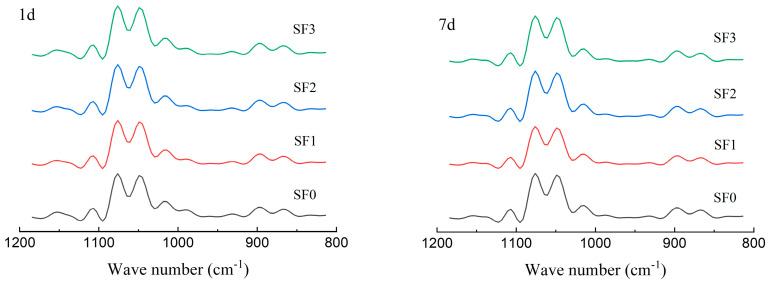
FT–IR spectra of sorghum flour leavened pancakes with different particle sizes stored for 1 d and 7 d.

**Figure 9:**
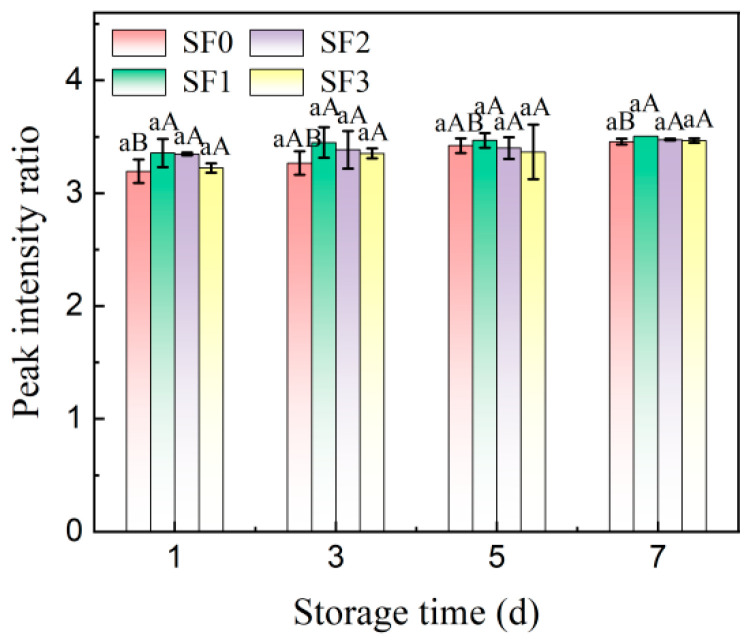
Changes in short–range order of starch molecules during storage of sorghum flour leavened pancakes with different particle sizes. SF0: wheat flour leavened pancake; SF1: sorghum flour leavened pancake with SF1 added; SF2: sorghum flour leavened pancake with SF2 added; SF3: sorghum flour leavened pancake with SF3 added. Different lowercase letters indicate significant differences between different treatments at the same storage time (*p* < 0.05). Different capital letters indicate significant differences in the same treatment at different storage times (*p* < 0.05).

Fourier transform infrared spectroscopy (FT–IR) can be used to observe the short–range molecular structure of starch granules. The typical absorption bands of starch crystal structures are 1047 cm^−1^ and 1022 cm^−1^, which represent the crystalline region and amorphous region, respectively. The ratio of peak intensity can be used as an index to measure the short–range order of starch molecules [35]. A lower ratio of 1047 cm^−1^ to 1022 cm^−1^ indicates a decreased degree of short–range order in starch crystallinity. Figure 8 shows the FT–IR spectra of sorghum flour leavened pancakes in the wavelength range of 800–1200 cm^−1^, and Figure 9 shows the changes in absorbance (R_1047/1022_) of sorghum flour leavened pancakes with different particle sizes during cold storage.

As shown in the figure, there was no significant change in the absorption peaks of each wavelength in the FT–IR spectra of the samples stored for 1 day and 7 days, indicating that no new functional groups were formed in the starch structure of the leavened pancakes during cold storage. With the increase in storage time, the R_1047/1022_ value of sorghum flour leavened pancakes increased, indicating that starch molecules rearranged during cold storage, thus forming a more orderly structure and strengthening the degree of aging. At the same time, with the decrease in the particle size of sorghum flour, the R_1047/1022_ value gradually decreased, indicating that the smaller particle sizes of sorghum flour would reduce the short–range order of starch molecules in the leavened pancakes. After 7 days of cold storage, the peak intensity ratio of the four groups of leavened pancakes increased by 8.23%, 7.54%, 4.44%, and 3.82%, respectively. It can be seen that with the decrease in sorghum flour particle size, the aging rate of leavened pancakes slowed down.

## 4. Conclusions

In this study, alterations in the quality of sorghum flour leavened pancakes, varying in particle size, were examined during their storage at cold temperatures. It was observed that the hardness of all the leavened pancakes increased substantially with the duration of storage. After seven days of refrigeration, the hardness of the wheat flour leavened pancake increased by 56.60%, indicating a rapid deterioration in quality. In contrast, the hardness of the sorghum flour leavened pancakes increased by 17.19%, 13.92%, and 12.27% for different particle sizes, respectively, which indicated a slower aging rate. Concurrently, as the storage period was extended, moisture migration occurred within the leavened pancakes, leading to a decrease in their moisture content. The change rates of A_21_ of wheat flour moisture and sorghum flour moisture with different particle sizes were 4.05%, 6.86%, 3.58%, and 3.29%, respectively. The sorghum flour with smaller particle sizes showed better water holding capacity. The aging of sorghum flour leavened pancakes during storage resulted in increased retrogradation enthalpy and crystallinity. However, a reduction in the particle size of sorghum flour mitigated these effects. Smaller particles of sorghum flour were found to impede the retrogradation of amylopectin, thereby slowing the aging process. Fourier transform infrared analysis indicated stable absorption peaks across various wavelengths during cold storage. Over time, the short–range orderliness of starch increased, but this was counteracted by smaller sorghum flour particles. The primary contributors to the aging of the leavened pancakes were starch retrogradation and moisture migration. As the particle size decreased, the water–holding capacity of the pancakes improved, inhibiting moisture migration and amylopectin recrystallization. This led to delayed aging and enhanced quality stability during storage. The results of this study not only expanded the varieties of coarse cereal leavened pancakes, but also offered a substantial experimental and theoretical foundation for enhancing the quality and storage longevity of leavened pancakes. Furthermore, it provided a theoretical groundwork for the application and exploitation of sorghum flour in flour–based foods and the industrial production of leavened pancakes. Future research will focus on gluten protein changes during storage and the impact of alternative pretreatment methods such as extrusion, microwave, and heat treatment on the quality of the leavened pancakes.

## Figures and Tables

**Table 1:** Particle size distribution of sorghum flour.

Sorghum Flour with Different Particle Sizes	D_10_ (μm)	D_50_ (μm)	D_90_ (μm)	D_(4, 3)_ (μm)
SF1	10.56 ± 0.08 ^a^	53.37 ± 1.45 ^a^	396.80 ± 2.97 ^a^	139.25 ± 1.20 ^a^
SF2	9.49 ± 0.10 ^b^	31.93 ± 0.60 ^b^	134.40 ± 3.39 ^b^	56.55 ± 1.51 ^b^
SF3	6.80 ± 0.00 ^c^	20.55 ± 0.01 ^c^	40.07 ± 0.05 ^c^	22.56 ± 0.01 ^c^

Different lowercase letters of the same index indicate significant difference (*p* < 0.05).

**Table 2:** Changes in moisture state of sorghum flour leavened pancakes with different particle sizes during storage.

Storage Time (d)	Groups	Relaxation Time (ms)		Proportion of Peak Area (%)	
		T_21_	T_22_	A_21_	A_22_
1	SF0	0.38 ± 0.02 ^abA^	9.66 ± 0.00 ^A^	23.71 ± 0.05 ^abA^	76.29 ± 0.05 ^bcC^
	SF1	0.33 ± 0.06 ^bA^	9.01 ± 0.00 ^A^	24.33 ± 0.04 ^aA^	75.67 ± 0.03 ^cC^
	SF2	0.41 ± 0.02 ^abA^	9.66 ± 0.00 ^A^	23.19 ± 0.71 ^bcA^	76.81 ± 0.71 ^abA^
	SF3	0.46 ± 0.04 ^aA^	10.35 ± 0.00	22.19 ± 0.19 ^cA^	77.81 ± 0.19 ^aA^
3	SF0	0.30 ± 0.00 ^cB^	9.01 ± 0.00 ^aB^	23.33 ± 0.01 ^aA^	76.67 ± 0.01 ^bBC^
	SF1	0.29 ± 0.01 ^cA^	8.13 ± 0.40 ^bB^	23.37 ± 0.07 ^aB^	76.56 ± 0.11 ^bAB^
	SF2	0.37 ± 0.00 ^aB^	9.01 ± 0.00 ^aB^	22.51 ± 0.00 ^bAB^	77.49 ± 0.00 ^aA^
	SF3	0.32 ± 0.00 ^bB^	9.01 ± 0.00 ^a^	21.94 ± 0.57 ^bA^	78.05 ± 0.56 ^aA^
5	SF0	0.28 ± 0.00 ^B^	8.71 ± 0.43 ^aB^	22.58 ± 0.14 ^abB^	77.42 ± 0.14 ^bcA^
	SF1	0.24 ± 0.00 ^AB^	7.84 ± 0.00 ^bBC^	23.09 ± 0.06 ^aB^	76.89 ± 0.08 ^cA^
	SF2	0.28 ± 0.00 ^C^	8.71 ± 0.30 ^aB^	22.41 ± 0.44 ^bcAB^	77.59 ± 0.44 ^abA^
	SF3	0.30 ± 0.00 ^B^	9.01 ± 0.00 ^a^	21.81 ± 0.09 ^cA^	78.19 ± 0.09 ^aA^
7	SF0	0.24 ± 0.00 ^bC^	7.84 ± 0.00 ^C^	22.75 ± 0.39 ^abB^	77.25 ± 0.39 ^bcAB^
	SF1	0.19 ± 0.01 ^cB^	7.32 ± 0.00 ^C^	23.46 ± 0.29 ^aB^	76.48 ± 0.24 ^cB^
	SF2	0.24 ± 0.01 ^bD^	8.41 ± 0.00 ^B^	21.60 ± 0.73 ^bB^	77.68 ± 0.30 ^bA^
	SF3	0.29 ± 0.01 ^aB^	8.41 ± 0.00	21.46 ± 0.26 ^bA^	78.54 ± 0.26 ^aA^

SF0: wheat flour leavened pancake; SF1: sorghum flour leavened pancake with SF1 added; SF2: sorghum flour leavened pancake with SF2 added; SF3: sorghum flour leavened pancake with SF3 added. Different lowercase letters indicate significant differences between different treatments at the same storage time (*p* < 0.05). Different capital letters indicate significant differences in the same treatment at different storage times (*p* < 0.05).

**Table 3:** Aging kinetic parameters of sorghum flour leavened pancakes.

Groups	Avrami Exponent *n*	Regeneration Rate Constant k (d^−1^)	R^2^
SF0	1.141	0.058	0.994
SF1	1.026	0.123	0.998
SF2	2.001	0.043	0.955
SF3	2.380	0.032	0.935

## Data Availability

The original contributions presented in this study are included in the article. Further inquiries can be directed to the corresponding author.
